# Exploring the Oral Health Status of Children and Parental Perspectives Regarding Knowledge, Awareness, and Dental Health Practices in the Tribal Communities of Melghat: An Epidemiological Cross-Sectional Study

**DOI:** 10.7759/cureus.91623

**Published:** 2025-09-04

**Authors:** Swanandi M Kelkar, Preetam P Shah, Shweta M Chaudhary, Shweta Jajoo, Laxmi S Lakade, Chetana Jagtap

**Affiliations:** 1 Pediatric Dentistry, Bharati Vidyapeeth (Deemed to be University) Dental College and Hospital, Pune, IND

**Keywords:** community health, oral health, oral health status, parental awareness, tribal

## Abstract

Background and aim

Gaining insights into the oral health challenges and specific dental practices of the tribal population in Melghat is essential for developing effective prevention and treatment strategies. This study aims to lay the foundation for targeted interventions that can enhance the oral health and overall well-being of tribal children aged two to 14 years in the Melghat region of Amravati district. This study aimed to assess oral health, hygiene practices, and the effects of malnutrition on oral tissues in tribal children aged two to 14 years, as well as to evaluate parental knowledge and practices regarding children's oral health in Melghat, a remote tribal area.

Materials and methods

The study included 1,023 tribal children aged two to 14 years. The Decayed, Missing, and Filled Teeth (DMFT/def) and Hypomineralized Second Primary Molars (HSPM) indices were assessed for all participants, while the Simplified Oral Hygiene Index (OHI-S) was recorded for children in the four-10- and 10-14-year age groups. In addition to the dental examination of the children, a questionnaire was administered to assess parental knowledge, awareness, and practices related to their children's oral health. This questionnaire was completed by the parents with assistance from local translators. Data were analyzed using SPSS version 25.0. To compare groups, independent samples t-tests were utilized, and chi-square tests were conducted to evaluate differences in proportions. A p-value of less than 0.05 was considered statistically significant.

Results

It was observed that around 1,001 parents, that is, 98% of parents, did brush their child’s teeth with a toothbrush and paste. Nine hundred parents, that is, around 88% parents, brush their child’s teeth once a day. No significant difference was seen regarding actions taken when a child has a toothache (p > 0.05). The majority of parents, that is, around 767 parents, breastfeed their child for two years (75%). The mean DMFT was highest in children aged six to 10 years (5.23). Age-wise comparison of children with CI-S revealed that the mean CI-S at 6six to 10 years of age was 3.98, while the mean CI-S for 10-14 years was 3.65. The mean DI-S at six to 10 years of age was 4.35 and the standard deviation was 2.19, while the mean DI-S for 10-14 years was 5.01, and this difference was not statistically significant (P > 0.05). DI-S and CI-S scores showed minimal age variation. MIH was found in 24.2% of the children, with 72% showing mild forms and 28% showing severe cases, indicating that one in every four children had MIH, and among those, one in four experienced a severe form. HSPM was identified in 14.5% of the children, with 91.7% of cases being mild and 8.3% severe. High prevalence of dental attrition and fluorosis was observed within this community.

Conclusion

Tribal children in Melghat face severe oral health challenges, including high DMFT, dental fluorosis, poor oral hygiene, and MIH. While some parental awareness exists, significant gaps remain, especially in diet and preventive care. Raising awareness and implementing targeted interventions on diet, prevention, and oral hygiene are crucial to improving oral health outcomes.

## Introduction

The term Adivasi refers collectively to a diverse range of ethnic and tribal groups considered to be the original inhabitants of India. These communities form a significant indigenous minority within the country. India is home to approximately half of the world’s indigenous population, encompassing 635 tribal communities, including 75 classified as particularly vulnerable tribal groups. According to the 2011 census [1], tribal communities make up 8.61% of India's total population, amounting to over 84 million individuals. For generations, their way of life and working conditions have been marginalized and suppressed by the dominant sections of society.

These populations reside in geographically isolated regions with limited access to essential resources, including balanced nutrition and safe drinking water. Furthermore, the absence of adequate healthcare infrastructure severely restricts their access to preventive and curative medical and dental services, contributing to a higher burden of disease. One of the primary challenges faced by tribal communities of late is the incorporation of sweets into their daily diets [2]. This has had a detrimental effect on the oral health of the children of such communities. Understanding the extent of the issues regarding oral health and the specific dental practices followed by the tribal population of Melghat is crucial for devising effective preventive and treatment strategies. Therefore, this study was undertaken to evaluate the oral health status, hygiene practices, and the impact of malnutrition on oral health among tribal children aged two to 14 years, along with assessing parental awareness and involvement. We hypothesized that poor nutritional status would be associated with a higher prevalence of dental caries and poorer oral hygiene. The two-14-year age range was chosen as it represents a critical period for the development of both dentition and long-term oral health behaviors. By conducting this study, we are trying to pave the way for targeted interventions that can improve the oral health and overall well-being of tribal children aged between two and 14 years, in the Melghat area, Amravati district.

## Materials and methods

Study design and subjects 

**Figure 1:**
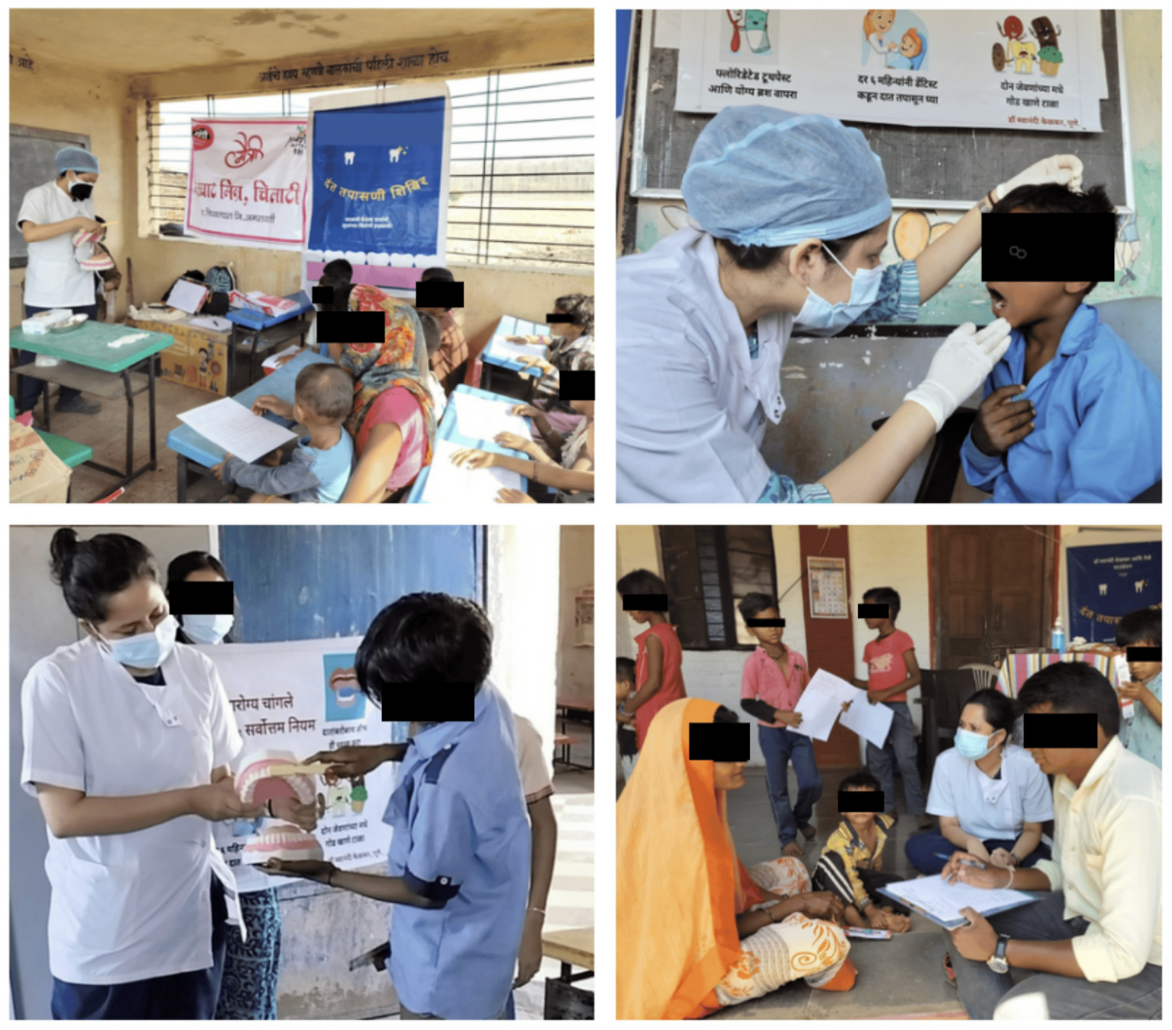
Demonstration of brushing technique, dental check-up in progress, and parents completing the questionnaire with a translator’s help.

The study was approved by the Institutional Ethics Committee of Bharati Vidyapeeth (Deemed to be University) Dental College and Hospital, Pune, abiding by the Helsinki Declaration of 1975, as revised in 2000. Written informed consent was obtained from the parents of the participants. A total of 1,023 children aged between two and 14 years who were residents of the tribal area of Melghat participated in the study. The children were grouped into the following age categories: two to four years, four to 10 years, and 10 to 14 years. The participants were informed about the dental check-up procedure. A translator was assigned to help communicate with the participants, as the local language spoken in Melghat is Korku, along with Hindi and Marathi. A brief case history of each participant was recorded. DMFT/def (Decayed, Missing, Filled Teeth Index) and HSPM index (Hypomineralized Second Primary Molar Index) were recorded in each participant. The OHI-S index (Oral Hygiene Index-Simplified) was recorded in the age groups of four to 10 years and 10-14 years. Along with the dental check-up of the children, a questionnaire regarding knowledge, awareness, and practices toward their children’s oral health was filled out. The questionnaire was filled out by the parents with the help of the local translators. The face and content validity of the questionnaire were evaluated by a panel of subject matter experts to ensure clarity, relevance, and appropriateness of the items. Oral hygiene instructions were given, and diet counselling was done. The brushing technique was demonstrated using models to provide clear, hands-on guidance for better understanding (Figure 1). 

Clinical examination

The clinical examination was conducted under standardized conditions to evaluate oral hygiene, dental caries experience, and enamel hypomineralization using the OHI-S [3], DMFT/def index [3], and HSPM index [4]. The OHI-S was used to assess oral hygiene by measuring debris and calculus levels. Six index teeth (#16, #11, #26, #36, #31, and #46, or their primary tooth equivalents) were examined on both buccal and lingual surfaces under adequate illumination. Each surface was scored from 0 to 3 for debris and calculus, with the combined scores averaged to obtain the OHI-S. For caries assessment of the permanent teeth, the DMFT index and for primary teeth def index were employed. The presence of decayed, missing due to caries, and filled teeth was recorded during a clinical examination using a mouth mirror and explorer, ensuring adherence to infection control protocols [3]. The evaluation of hypomineralization in second primary molars was carried out using the HSPM index, following the guidelines set by the European Academy of Paediatric Dentistry (EAPD) [4]. Every second primary molar was inspected for signs of hypomineralization defects, which were categorized as mild (discoloration without enamel loss), moderate (discoloration with partial enamel loss), or severe (extensive enamel loss or post-eruptive breakdown). All findings were systematically recorded in a pre-designed data collection form.

Statistical analysis

Data entry was performed using Microsoft Excel 2009 (Microsoft Corp., USA). Descriptive analyses such as frequencies, means, and standard deviations were computed. To compare groups, independent samples t-tests were utilised, and chi-square tests were conducted to evaluate differences in proportions. An independent t-test was utilised for comparison of DI-S and CI-S between males and females. A p-value of less than 0.05 was considered statistically significant. All statistical procedures were carried out using IBM SPSS Statistics for Windows, version 25.0 (released 2017, IBM Corp., Armonk, NY). 

## Results

Regarding the parents getting information about their child's oral health, it was found that among the various sources reported, the majority were PHCs (primary health centers), with 95%, followed by dentists in 3%. Meanwhile, the lowest weightage was given to an Anganwadi worker.

The questionnaire provided to the parents was divided into two sections: one focusing on awareness and the other on practices, as mentioned (Table 1).

**Table 1:** The questionnaire provided to the parents was divided into two sections: one focusing on awareness and the other on practices, as mentioned.

Category	Questions	Options
Knowledge	From where do you get information about dental health?	
	Do you think baby teeth are important?	Yes / No / Don’t Know
	Do you think there is a need for a dental surgeon/dentist in your area?	Yes / No / Don’t Know
	Do you think oral health is important for maintaining a child's overall health?	Yes / No / Don’t Know
	Do you think diet affects a child's dental health?	Yes / No / Don’t Know
	Are you aware that a mother’s oral health influences the oral health of her child.	Yes / No / Don’t Know
Practices	Do you assist your child with brushing their teeth? If yes, could you describe how you brush your child's teeth? If not, what method does your child use to clean their teeth?	Yes / No / Don’t Know Toothbrush & paste / Ayurvedic powder / Stick of tree / Just water / Other Toothbrush & paste / Ayurvedic powder / Stick of tree / Just water / Other
	How often do you brush your child's teeth?	Once a day / Twice a day / After meals / Not regular
	What actions do you take if your child has a toothache?	
	Is your child exposed to sweet foods?	Yes / No / Don’t Know
	Until what age did you breastfeed your child?	A year / Two years / Three years / Other
	Do you follow any customs/traditions regarding your baby's diet or oral health after birth?	

From the responses of the respondents, it was observed that around 60% of parents were aware that baby teeth are important, and 70% knew that there is a need for a dentist in the local area. Moreover, 63% knew that a child’s oral health is important for overall health, while 74% parents did not know that diet affects child’s dental health, 86% did not know that child's first permanent tooth erupts at the age of six, and 81% were unaware that a mother’s oral health plays a significant role in influencing the oral health of her child (p < 0.05*).

Responses to the questionnaire for Awareness and Practice are shown in Table 2 and Table 3, respectively. 

**Table 2:** Response to the questionnaire for Awareness. Statistical analysis was done by using a Chi-square test.

Questions	Options	Frequency	Percentage	Chi square	P value
Do you think baby teeth are important	Yes	614	60	31.84	p<0.001**
	No	61	6		
	Don’t know	348	34		
Do you think there is a need for a dental surgeon/Dentist in your area?	Yes	716	70	52.83	p<0.001**
	No	173	17		
	Don’t know	132	13		
Do you think oral health is important for maintaining a child's overall health?	Yes	634	62	22.39	p<0.001**
	No	215	22.5		
	Don’t know	174	17.5		
Do you think diet affects a child's dental health?	Yes	102	10	31.54	p<0.001**
	No	757	74		
	Don’t know	164	16		
Did you know that a child's first permanent tooth erupts at the age of six?	Yes	143	14	29.23	p<0.001**
	No	880	86		
	Don’t know	0	0		
Did you know that a mother's oral health influences the oral health of her child.	Yes	194	19	17.25	p<0.001**
	No	829	81		
	Don’t know	0	0		

**Table 3:** Response to the questionnaire for Practice. Statistical analysis was done using a chi-square test.

Questions	Options	Frequency	Percentage	Chi square	P value
Until what age did you breastfeed your child?	A year	167	16	37.19	p<0.001**
	Two years	767	75		
	Three years	82	8		
	Other	10	1		
Do you brush your child's teeth?	Yes	1001	98	31.84	p<0.001**
	No	20	2		
	Don’t know	0	0		
If yes, How?	Toothbrush and toothpaste	808	79	52.83	p<0.001**
	Ayurvedic powder	72	7		
	stick of tree	31	3		
	just water	51	5		
	Other:	61	6		
If not, how does the child clean their teeth?	Toothbrush and toothpaste	250	24.4	22.39	p<0.001**
	Ayurvedic powder	435	42.5		
	stick of tree	105	10.3		
	just water	131	12.8		
	Other:	102	10		
How often do you brush your child's teeth?	Once a day	900	88	42.87	p<0.001**
	Twice a day	51	5		
	After the meal	41	4		
	Not regular	31	3		
What actions do you take if your child has a toothache?	Doctor/PHC	483	47	42.87	p>0.05
	Hot water with salt gargles	174	19		
	Ayurvedic powder	61	6		
	Jadibuti	183	18		
	None	102	10		
Is your child exposed to sweet foods?	Yes	808	79	12.42	p<0.001**
	No	215	21		
	Don’t know	0	0		
Do you follow any customs/traditions regarding baby's diet or oral health after birth?	Use of teak charcoal	194	19	09.23	p<0.001**
	Not any	829	81		

**Figure 2:**
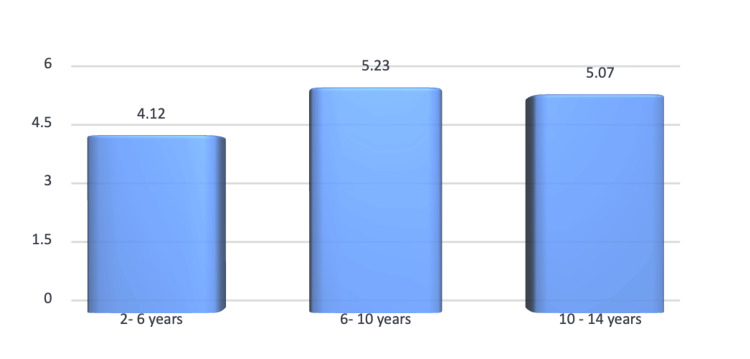
Age-wise distribution of the prevalence of DMFT DMFT: Decayed, Missing, Filled Teeth Index

It was observed that around 1,001 parents, that is, 98% of parents, did brush their child’s teeth with a toothbrush and paste. Nine hundred parents, that is, around 88% parents, brush their child’s teeth once a day. No significant difference was seen regarding actions taken when a child has a toothache (p > 0.05). The majority of parents, that is, around 767 parents, breastfeed their child for two years (75%), and a majority of them, that is, 829 parents (81%), did not follow any custom or traditions on oral health after birth (p < 0.05*). Age-wise comparison of children with DMFT was made. The mean DMFT at two to six years of age was 4.12, and the standard deviation was 2.12. Meanwhile, the mean DMFT for six to 10 years and 10-14 years was 5.23 and 5.07, and this difference was not statistically significant (p > 0.05). The age-wise distribution of the prevalence of DMFT is presented in Figure 2.

**Figure 3:**
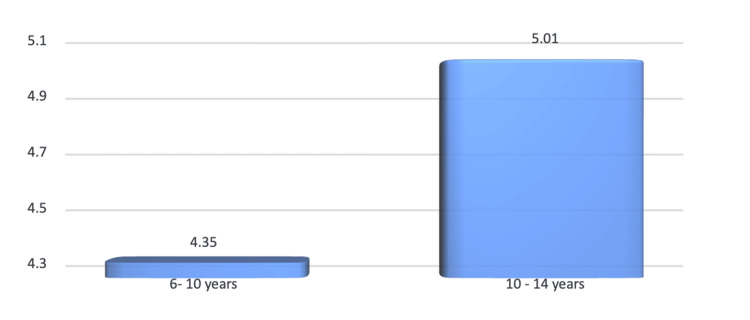
Age-wise distribution of prevalence of DI-S DI-S: Debris Index Simplified

Age-wise comparison of children with DI-S was made. The mean DI-S at six to 10 years of age was 4.35 and the standard deviation of 2.19, while the mean DI-S for 10-14 years was 5.01, and this difference was not statistically significant (p > 0.05) (Figure 3).

The gender-wise analysis of DI-S and CI-S scores showed that males had a mean DI-S score of 4.21 (SD = 4.20), while females recorded a mean of 3.98 (SD = 4.12), with no statistically significant difference between the groups (p > 0.05). Similarly, for CI-S, the average score for males was 4.65 (SD = 2.20), and for females, it was 4.12 (SD = 1.12), again indicating no significant gender-based difference (p > 0.05). An independent t-test was utilised for comparison of DI-S and CI-S between males and females.

**Figure 4:**
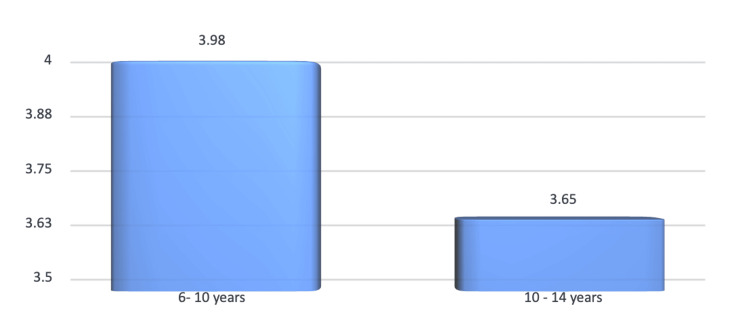
Age-wise distribution of prevalence of CI-S CI-S: Calculus Index Simplified

Age-wise comparison of children with CI-S was made. The mean CI-S at six to 10 years of age was 3.98, while the mean CI-S for 10 - 14 years was 3.65, and this difference was not statistically significant (p > 0.05) (Figure 4).

Miscellaneous findings

**Figure 5:**
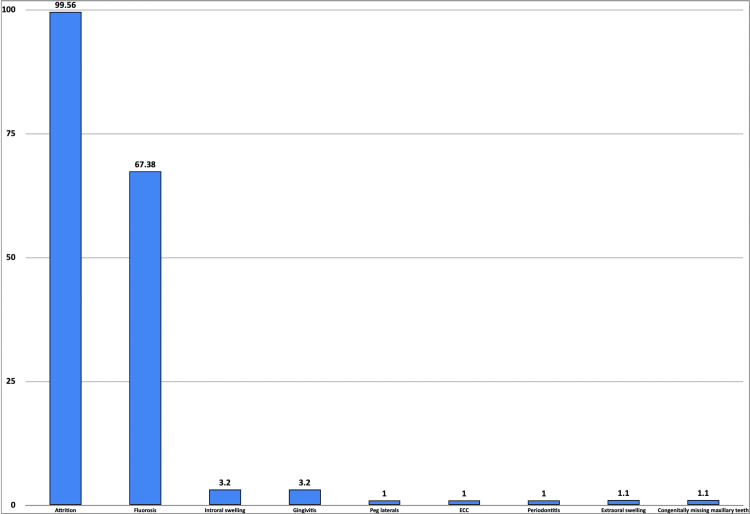
A bar diagram demonstrating the miscellaneous findings Y-axis: percentage of subjects (%), X-axis: type of clinical finding

A variety of miscellaneous findings were observed, as shown in Figure 5. The majority of cases had attrition, followed by fluorosis. 

MIH was found in 24.2% of the children, with 72% showing mild forms and 28% showing severe cases, indicating that one in every four children had MIH, and among those, one in four experienced a severe form. HSPM was identified in 14.5% of the children, with 91.7% of cases being mild and 8.3% severe (Table 4). Affected molars and incisors occurred together more often than molars alone. On average, 2.77 permanent first molars, 1.29 permanent incisors, and 1.96 second primary molars were involved. Maxillary teeth were more commonly affected than mandibular teeth.

**Table 4:** Prevalence of hypomineralized second primary molars and molar incisor hypomineralization. MIH: molar incisor hypomineralization, HSPM: hypomineralized second primary molars

Parameters	Prevalence	Mild	Severe
Molar incisor hypomineralization (MIH)	24.2%	72%	28%
Hypomineralized second primary molars (HSPM)	14.5%	91.7%	8.3%

In the study, the first primary molars were the most commonly affected teeth, with a mean of 2.77 affected per child (2.27 mild, 0.5 severe). The second primary molars followed, with a mean of 1.96 (1.86 mild, 0.1 severe), while incisors had a lower mean of 1.29, all mild. Regarding the number of teeth affected, 35% of children had four or more affected first molars, compared to 26% with three, 22% with two, and 17% with one. Among incisors, 25% had one affected, while 12%, 13%, and 10% had two, three, and four or more, respectively. For the second primary molars, 35% had one affected, 41.7% had two, 15% had three, and 8.3% had four or more.

Tooth-wise prevalence showed the highest caries rates in the first primary molars: 16 (18.6%), 26 (17.8%), 36 (14.5%), and 46 (15.9%). Incisor involvement was the highest in tooth 11 (8.7%) and tooth 21 (6.5%), with other incisors ranging from 1.9% to 3.4%. Among the second primary molars, prevalence was 8.9% in 55, 7.5% in 65, 3.9% in 75, and 6.8% in 85.

## Discussion

Parental awareness and attitudes 

The majority of parents (95%) relied on PHCs for information about their children’s oral health, with dentists contributing only 3%. This reliance mirrors findings by Goel et al. (2019) [5] and Das et al. (2023) [6], who emphasized the critical role of community health resources in rural areas. However, significant knowledge gaps were observed; 74% of parents were unaware of the impact of diet on dental health, and 86% did not know the eruption age of permanent teeth. These deficits are consistent with findings by Nepaul P et al. (2020) [7], highlighting the need for targeted oral health education initiatives. Approximately 60% of parents acknowledged the importance of milk teeth, consistent with Dhirja et al. [8]. However, only 10% recognized the role of diet in caries prevention, mirroring Kamolmatyakul et al. [9]. Furthermore, 63% understood the relationship between oral and overall health, similar to Abhinava et al.'s observations [2].

Practices regarding oral health 

Encouragingly, 98% of parents reported brushing their children’s teeth with a toothbrush and toothpaste, surpassing the 82% reported by Lago et al(2016) [10]. However, 88% brushed only once daily, indicating the need for reinforcement of twice-daily brushing practices, as recommended by the American Academy of Pediatric Dentistry. Breastfeeding for two years was common (75%), but any specific oral health traditions and customs after birth were not followed, similar to the findings by Raju et al. [11].

Prevalence of dental caries and oral hygiene indices 

The mean DMFT scores were 4.12 (two to six years), 5.23 (six to 10 years), and 5.07 (10-14 years), with no statistically significant differences across age groups (p > 0.05). These results align with studies by Maru et al. and Whelton et al. [12,13], but contrast with the lower DMFT values reported by Saravanan et al. [14] for similar populations. Oral hygiene indices (DI-S and CI-S) revealed no significant gender or age-based differences. However, the slightly higher DI-S score in the 10-14 years group suggests a decline in hygiene practices with age, consistent with Singh et al. [2].

Periodontal and nutritional influences 

Periodontal health deteriorated with age, reflecting findings by Singh et al. [2] and Villalobos et al. [15], and overall oral hygiene was poor, likely due to inadequate access to dental care and limited oral health awareness. Malnutrition during early development was associated with hypomineralized and fragile enamel, corroborating Alvaro et al.'s findings [16]. In addition, the prevalence of dental fluorosis was significantly higher among tribal children, potentially linked to nutritional deficiencies, as noted by Shanti et al. [17].

Enamel hypomineralization (MIH and HSPM) 

MIH was observed in 24.2% of children, with 28% exhibiting severe forms, aligning with global estimates by Elfrink et al. (2021) [18]. HSPM was noted in 14.5%, predominantly mild (91.7%), consistent with Chawla et al. and Garot et al. [19]. Maxillary teeth were more frequently affected, supporting findings by da Costa-Silva et al., indicating systemic developmental susceptibilities [20].

Limitations

Limitations include the reliance on self-reported data from parents, which may introduce recall bias. The cross-sectional design of the study limits the ability to establish causal relationships. Regular follow-up is crucial for studies conducted in tribal areas like Melghat to ensure sustained impact. Future studies should incorporate longitudinal designs to better capture changes in oral health practices and outcomes over time. 

## Conclusions

The translational value of our study lies in its community-based approach to improving pediatric oral health in an underserved tribal population. By combining clinical assessments (DMFT/def, HSPM, OHI-S) with parental knowledge and practice surveys, the study offers actionable insights into both clinical and behavioural aspects of oral health. Importantly, this study extended beyond data collection by providing immediate health benefits to the community through personalized oral hygiene instructions, dietary counseling, and hands-on brushing technique demonstrations. This approach bridges the gap between research and real-world impact, emphasising the study’s strong translational value and its potential to inform future public health interventions in similar rural and tribal regions. The findings can help inform culturally sensitive oral health programs, guide training of local health workers, and support public health planning for similar linguistically and geographically marginalised communities. 

Despite some parental awareness, critical gaps remain in understanding the importance of diet, preventive care, and timely dental interventions. The findings underscore the urgent need for targeted public health initiatives aimed at improving oral hygiene habits, educating parents on proper nutrition and preventive care, and promoting early dental visits. Addressing these issues could lead to significant improvements in the oral health and overall well-being of children in these marginalised communities.
